# Pediatric quality of life multidimensional fatigue scale (PedsQL-MFS) detects the effects of a 3-week Inpatient body weight reduction program for children and adolescents with obesity

**DOI:** 10.1186/s12955-021-01907-5

**Published:** 2022-01-10

**Authors:** Matthew F. Smout, Gian Mauro Manzoni, Sofia Tamini, Nicoletta Marazzi, Alessandra De Col, Giada Pietrabissa, Gianluca Castelnuovo, Enrico Molinari, Alessandro Sartorio

**Affiliations:** 1 Istituto Auxologico Italiano IRCCS, Experimental Laboratory for Auxo-Endocrinological Research,, Milan and Piancavallo, VB Italy; 2 Justice and Society, University of South Australia, Adelaide, South Australia Australia; 3 Psychology Research Laboratory, Istituto Auxologico Italiano IRCCS, Milan and Piancavallo, VB Italy; 4 Faculty of Psychology, eCampus University, Via Isimbardi 10, 22060 Novedrate, Como, Italy; 5 Department of Psychology, Catholic University of Milan, Milan, Italy; 6 Division of Auxology and Metabolic Diseases, Istituto Auxologico Italiano IRCCS, Piancavallo, VB Italy

**Keywords:** Pediatric obesity, Fatigue, Multidimensional fatigue scale, Body weight reduction program

## Abstract

**Background:**

Fatigue is a frequent complaint amongst children and adolescents with obesity, and it interferes with adherence to dietary and exercise regimes that could reduce obesity. The present study evaluated the effect of an inpatient 3-week body weight reduction program on body weight and fatigue.

**Method:**

One hundred children and adolescents with obesity (64% female; aged 11–18 years) undertook an inpatient program of personalized diet, daily exercise, education, and counselling.

**Results:**

The sample evidenced a mean reduction in body mass (females: *ΔM* = 4.3 (sd = 2.1) kg, *p* < .001), males: *ΔM* = 6.2 (sd = 2.6) kg, *p* < .001), BMI standard deviation score (females: *ΔM* = 0*.*17 (sd = 0.07), males: *ΔM* = 0*.*24 (sd = 0.08), *p* < .001) and fatigue (females: *ΔM* = 7.8 (sd = 9.7), males: *ΔM* = 5*.*0 (sd = 6.9), *p* < .001) as measured by the Pediatric Quality of Life Multidimensional Fatigue Scale (PedsQL-MFS) and improvements on the Attention problems subscale of the Youth Self Report (total sample: *ΔM* = 0*.*89 (sd = 2.44), *p* < .001). Reliable change analyses revealed fatigue changes were achieved by up to 34% females and 17% males, but the majority did not achieve reliable change and changes in fatigue were not correlated with changes in body mass.

**Conclusions:**

The program achieved clinically significant improvements in some children and adolescents. Future studies should explore predictors of treatment responsiveness.

*Trial registration* Observational study. Not registered.

## Introduction

The prevalence of obesity globally has nearly tripled between 1975 and 2016, with the prevalence of overweight and obesity among children and adolescents increasing from 4 to 18% [69]. In children, rates of both obesity and its co-morbidities are rising all over the world [14], making preventing and reducing obesity a critically important social and medical necessity. Children and adolescents who are obese are five times more likely to be obese in adulthood [55] and childhood obesity is a risk factor for a number of comorbid medical conditions. Obesity is the main risk factor for type 2 diabetes in children [7]; children with obesity are four times likely to develop type 2 diabetes than children of normal weight [1]. Rates of obstructive sleep apnoea in children with obesity have varied between 24 and 61% [6]. Between 67–86% children with severe obesity evidence at least one cardiometabolic risk factor [7]. These include dyslipidemia, such as increased triglycerides (TG), free fatty acids, and reduced high-density lipoproteins, hypertension and glycated hemoglobin. Approximately 23.6% of Italian children and adolescents with obesity suffer from metabolic syndrome [12], the prevalence being even higher in BMI- and age- matched Brazilian (34.8%, 11] and German (40.4%) children/adolescents [54]. Higher childhood BMI is associated with hyperinsulinemia during childhood, which prospectively predicts metabolic syndrome in adulthood [70]. The impact of childhood obesity is not restricted to physical health, as childhood obesity is associated with low self-esteem [24], poor body image [25], depression [67] and decreased cognitive performance [38].

Effective weight-management programs in children and adolescents involve multiple techniques and strategies including dietary therapy, physical activity, and behavior therapy to foster long-term weight control and prevention of weight regain. Lifestyle interventions involving either dietary, physical activity or behavioural changes, or their combination, have been reported to produce weight loss and improvements in LDL, TG, fasting insulin and blood pressure of children with obesity [27]. Very low energy diet programs, typically limited to ≤ 800 kCal per day or < 50% energy expenditure which last between 3–20 weeks have been found to achieve a mean weight loss of 10.1 kg [5]. However, these programs have been criticized for potentially impairing children’s growth [3, 8, 16] frequent inability of patients to adapt to this demanding approach and increased risk of recidivism when the very low energy diet is discontinued [34]. Increasing physical activity alone has sometimes been found to reduce BMI in children who are overweight [21, 68]. The majority of programs incorporate multiple components in outpatient settings, but concerns remain that the amount of change in BMI achieved is rarely enough to produce improvements in cardiovascular risk factors [20, 42].

A growing number of inpatient multicomponent programs have produced high weight loss and improvements in cardiovascular risk factors [10, 52]. Recently, short-stay programs have been demonstrated to achieve comparable improvements to programs which previously typically took 6–12 months [40, 48], reducing the costs associated with treatment. Recently, two studies have demonstrated that a 3-week multidisciplinary body weight reduction program (BWRP), entailing energy restricted diet, daily physical activity, psychological counselling and nutritional education achieved significant reductions in body mass (BM) and fat mass (FM), and improvements in lower limb muscle power, and fatigue [36, 51].

Improvements in fatigue may be a particularly important outcome of weight loss programs. Fatigue has been defined as extreme, persistent tired, weakness or exhaustion during physical or intellectual activity that is not reduced or alleviated by resting [17, 44]. Fatigue is associated with significant distress and psychological disability and is a frequent reason for adolescents presenting to primary medical services [13, 43]. Fatigue is frequently among the most distressing symptoms associated with a range of paediatric medical conditions [26] including cancer [46], primary immunodeficiencies [50], paediatric-onset multiple sclerosis [57], lupus [18], and juvenile idiopathic arthritis [66] contributing to poorer health-related quality of life [19]. Tasks that produce fatigue have been demonstrated to increase pain in people with fibromyalgia [15]. Fatigue reduces energy levels, mood, motivation, interferes with concentration and adversely affects relationships as the sufferer tends to cope via withdrawal from people and usual activities [53, 59]. Therefore, reducing fatigue is an important target in improving the quality of life for children with any medical condition.

Fatigue is common in obesity [28]. Children with obesity report elevated fatigue compared to healthy peers [64] and fatigue is associated with greater fat mass [62]. In addition to its direct contribution to poorer quality of life, fatigue also interferes with therapeutic activities that could otherwise alleviate it. Fatigue makes physical exercise more difficult [56] and impedes adherence to diets [45]. Conversely, reducing fatigue improves cognitive functioning [61], which, in turn, may facilitate cognitive reappraisal, which is also associated with successful outcomes of weight loss programs [49]. Therefore, fatigue is an important indicator of the impact of intervention programs for paediatric obesity.

A previous evaluation [51] of the present 3-week inpatient program found improvements in fatigue measured by the Fatigue Severity Scale (FSS, [28]. This finding was encouraging and warrants replication with other measures of fatigue to verify that it is robust. The FSS is a unidimensional measure that focus on functional impact or impairment due to fatigue. Although the FSS often correlates highly with other measures of fatigue, different measures have demonstrated superior performance in some populations. For example, in a study of patients with Multiple Sclerosis, the FSS correlated more strongly with the Physical subscale than the Cognitive subscale of the Modified Fatigue Impact Scale (MFIS) and the MFIS demonstrated superior precision in measuring high levels of fatigue [4]. The PedsQL-MFS has been increasingly used with pediatric populations [46, 58] and offers the possibility of detecting differential responses to interventions in fatigue across cognitive, physical and sleep/rest dimensions. The purpose of this study is to see the extent to which the 3-week body weight reduction program tested by Rigamonti et al. [51] program produces improvement across dimensions in fatigue as measured by the Pediatric Quality of Life Multidimensional Fatigue Scale (PedsQL-MFS) [63].

## Methods

### Participants

One hundred children and adolescents with obesity (64 girls, 36 boys; age *rng* = 11–18 years, *M* = 15.34, *SD* = 1.61) participated in this study. The majority had achieved secondary school education (78%).

Obesity was defined as having a BMI standard deviation score (BMI SDS) for gender and chronological age greater than 2, according to the Italian growth charts [11].

Participants were recruited as in-patients from the Division of Auxology, Istituto Auxologico Italiano IRCCS, Piancavallo (VB) Italy. All subjects had a full medical history and physical examination, including routine hematology and biochemistry screens and urine analysis. In terms of comorbidities, 2 children were receiving treatment for respiratory diseases, 2 children had orthopedic problems but were not receiving treatment for these, one child was receiving treatment for a neurological disorder, 6 had endocrinological diseases–2 of whom were receiving treatment for hypothyroidism, 2 for diabetes and 2 children were not receiving treatment. All the comorbidities were adequately controlled.

### Measures

#### ***Pediatric quality of life inventory***–***multidimensional fatigue scale (PedsQL-MFS)***

The PedsQL-MFS is a questionnaire consisting of 18 items describing symptoms of fatigue. Each item is rated for how frequently it is a problem on a 5-point scale from 0 “almost never” to 4 “almost always”. Italian child-report versions for 8–12 year-olds and 13–18 year-olds previously validated for use in pediatric obesity by our group were used [41]. In this study, internal consistency at baseline (α = 0.88) and test–retest reliability for the total score between admission and day 3 scores was high (*r* = 0.85) with no significant differences in scores between these occasions (*t*(97) = 1.54, *p* = 0.127). Items were designed to represent 3 × 6-item subscales: Sleep/Rest Fatigue (*e.g.,* “I sleep a lot”); Cognitive Fatigue (“It is hard for me to keep my attention on things”) and General Fatigue (“I feel physically weak(not strong)”). In this study, values for internal consistency and test–retest reliability for subscales were: Sleep/Rest Fatigue α = 0.73 (acceptable) and *r* = 0.86 (good); Cognitive Fatigue α = 0.87 (good) and *r* = 0.86 (good); General α = 0.81 (good) and *r* = 0.78 (acceptable). Reliable change indices (RCIs) based on test–retest reliability and baseline standard deviations from this study (see Statistical Analysis for how these were calculated) were: Total = 11.96; Sleep-Rest = 4.90; General = 5.34; Cognitive Fatigue = 5.17. Although there is a parent proxy-report version, in the current study, only the self-report version was used.

#### Youth self-report (YSR)

The Italian version of the Youth Self-Report is a questionnaire containing 112 items that participants aged 11–18 rate on a 3-point scale: 0 “not true”; 1 “somewhat or sometimes true”; or 2 “very true or often true” [22]. It produces eight subscales: Anxious/Depressed (*e.g.,* “I am afraid of going to school”); Withdrawn/Depressed (*e.g.,* “There is very little that I enjoy”); Somatic Complaints (*e.g.,* “Headaches” (without known medical causes)); Social Problems (*e.g.,* “I don’t get along with other kids”); Thought Problems (*e.g.,* “I have thoughts that other people would think are strange”); Attention Problems (*e.g.,* “I act too young for my age”); Rule-Breaking Behaviour (*e.g.,* “I hang around with kids who get in trouble”); and Aggressive Behaviour (*e.g.,* “I am mean to others”). In this study, raw scores rather than T-scores are reported. RCIs were calculated using sample baseline standard deviations and test–retest reliability values for the English version [2]. RCIs and baseline internal consistency values in this sample were: Anxious/Depressed RCI = 7.55, α = 0.85; Withdrawn/Depressed RCI = 5.86, α = 0.83; Somatic Complaints RCI = 4.56, α = 0.70; Social Problems RCI = 5.19; α = 0.69; Thought Problems RCI = 4.98; α = 0.76; Attention Problems RCI = 3.51, α = 0.71; Rule-Breaking Behaviour RCI = 4.81; α = 0.82; Aggressive Behaviour RCI = 5.40, α = 0.85.

### Procedures

#### Body weight-reduction program (BWRP)

##### Diet and nutritional education

During the 3-week BWRP, personalized diets were offered according to the initial basal metabolic rate test and physical activity level for each patient. The calories to be introduced with the diet were calculated by subtracting approximately 25% from the value of resting energy expenditure as measured in each patient by indirect calorimetry (Vmax 29; SensorMedics Corporation, Yorba Linda, CA, United States) for a total duration of 20 min. Energy supply comprised 21% proteins, 53% carbohydrates, and 26% lipids. The composition of diet was formulated according to the Italian recommended daily allowances [47]. Each patient was free to choose foods from a heterogeneous daily menu. Foods to which the patient declared to be allergic were removed from the menu. Five daily portions of fruits and vegetables were mandatory. A fluid intake of at least 1500 mL/day was encouraged. Moreover, the dieticians’ team verified that each subject had finished every meal (all the subjects considered in the present study finished the 98% of the meals).

During the BWRP the patients had dietetics lessons consisting of lectures, demonstrations and group discussions with and without a supervisor, which took place every day throughout the whole rehabilitation period.

##### Psychological counseling

Cognitive behaviour therapy was provided by a clinical psychologist 2–3 times per week based in either individual or group sessions. Strategies taught included stimulus control procedures, problem solving and stress management training, development of healthy eating habits, assertiveness and social skills training, cognitive restructuring of negative maladaptive thoughts and relapse prevention training. Whenever possible (usually, 1 day per week), supplementary sessions were also performed with patients’ parents, aimed at improving motivation to change lifestyle and interpersonal communication.

##### Physical activity

During the 3-week BWRP, children and adolescents participated in a personalized exercise-training program, from Monday to Friday, under the guidance of a therapist. Training sessions lasted 45–60 min per day (preceded and followed by 5–7 min stretching) and were mainly made up of aerobic activities (walking on a treadmill or cycling on an ergometer) under heart rate (HR) monitoring and medical supervision. All subjects completed 15 sessions of physical training. The intensity of endurance exercises was set at a HR corresponding to 60 and 80% of the individual maximal HR estimated as 220-age (year). In addition, subjects had 1 h/day of aerobic leisure activities at the institution on Saturday and Sunday. The research assistant and the physical trainers verified that each subject participated in each training session, performed exercises correctly, and completed at least 95% of the exercise session and program. All study participants completed at least 96% of the exercise program.

### Data collection

Self-report measures were collected on day 1 (admission) and day 21 (discharge) of the inpatient program.

### Statistical analysis

There was no missing data. Overall changes in fatigue, BM, BMI and Youth Self-report were evaluated using paired t-tests and associated Cohen *d* effect sizes. Gender, age and BMI SDS as potential moderators were tested using General Linear Models with fatigue as a repeated measure. Data were approximately normal according to visual inspection of frequency histograms. Univariate outliers varied from 0 to 3 cases per analysis; in each case their inclusion or exclusion did not affect significance of comparisons, so were removed per analysis. The proportion of participants achieving reliable change on the PedsQL-MFS was calculated using the formula for a 95% reliable change confidence interval: RCI = standard error of change × 1.96 [29, 30]. Standard error of change (SE_DIFF_) was calculated as SE_DIFF_ = sqrt(2*SE^2^), where SE = SD_PRE_ * sqrt(1 – *r*_xx_). SD_PRE_ was our sample’s admission standard deviation value for the measure and *r*_*xx*_ is the published test–retest reliability of the measure. The RCI denotes the magnitude of difference between admission and discharge scores on a measure that a participant would need to obtain for there to be a < 5% probability that a difference of this size would be simply due to test–retest measurement error. RCI values were obtained using the online calculator at: https://www.psyctc.org/stats/rcsc1.htm. All analyses were conducted using SPSS 27.0

## Results

Tables 1, 2 reports raw means at admission and discharge (day 21) for BM, BMI SDS and PedsQL-MFS scores by gender (outliers included).

**Table 1:** Participant characteristics (*n* = 100)

Characteristic	
Gender [*n* (%)]	
Female	64 (64%)
Male	36 (36%)
Age [*M (SD)*]	15.34 (1.61)
Height (m) [*M (SD)*]	1.66 (0.09)
Body mass (kg) [*M (SD)*]	104.4 (19.0)
BMI SDS [*M (SD)*]	3.05 (0.51)
Highest education level completed [*n* (%)]	
Primary school	22 (22%)
Secondary school	78 (78%)
Occupation [*n* (%)]	
Employed	1 (1%)
Unemployed	3 (3%)
Secondary school	
First year	2 (2%)
Second year	17 (17%)
Third year	4 (4%)
High school	
First year	30 (30%)
Second year	22 (22%)
Third year	12 (12%)
Fourth year	9 (9%)

**Table 2:** Means and standard deviations for body mass and fatigue at admission and discharge by gender

Measure	Gender	Admission *M (SD)*	Discharge *M (SD)*	Paired-group t-test
BM (kg)	Females (n = 64)	99.0 (16.3)	94.7 (15.6)	*t*(63) = 16.84, *p* < .001
	Males (n = 36)	114.0 (19.7)	107.9 (17.9)	*t*(35) = 14.14, *p* < .001
BMI SDS	Females (n = 64)	3.01 (0.46)	2.84 (0.48)	*t*(63) = 17.75, *p* < .001
	Males (n = 35)	3.08 (0.52)	2.85 (0.52)	*t*(34) = 16.92, *p* < .001
Total fatigue	Females (n = 63)	23.5 (11.5)	15.6 (9.7)	*t*(62) = 6.41, *p* < .001
	Males (n = 35)	18.4 (9.1)	13.4 (9.3)	*t*(34) = 4.31, *p* < .001
Sleep/rest fatigue	Females (n = 63)	8.56 (4.86)	6.73 (4.50)	*t*(62) = 2.97, *p* = .004
	Males (n = 36)	6.94 (4.45)	5.81 (4.71)	*t*(35) = 2.10, *p* = .043
General fatigue	Females (n = 64)	7.94 (4.02)	5.00 (3.65)	*t*(63) = 5.76, *p* < .001
	Males (n = 36)	6.28 (4.11)	4.06 (3.60)	*t*(35) = 4.70, *p* < .001
Cognitive fatigue	Females (n = 63)	6.70 (5.24)	4.43 (4.37)	*t*(62) = 3.85, *p* < .001
	Males (n = 35)	5.91 (4.55)	4.40 (3.98)	*t*(34) = 2.21, *p* = .034

BM = Body mass; BMI SDS = Body mass index standard deviation scores

### Body weight

For the full sample there was a significant reduction in BM from day 0 to day 21 (*t*(98) = 20.5, p < 0.001) with a large effect size (*d* = 2.06, *95%CI*[1.71, 2.41]). Results varied by gender: males remained heavier throughout (*F*(1, 98) = 15.9, *p* < 0.001) and lost more weight over the 21 day program (*F*(1, 98) = 15.5, *p* < 0.001). They achieved a change in BM of *M* = 5.3% (*SD* = 1.7%), while females achieved a change of BM of *M* = 4.3% (*SD* = 1.8%).

For the full sample, there was a significant reduction in BMI SDS from day 0 to day 21 (*t*(98) = 22.6, *p* < 0.001) of large effect size (*d* = s 2.27, *95%CI*[1.89, 2.64]). There was no main effect of gender on BMI SDS (*F*(1, 97) = 0.16, *p* = 0.689), but males made greater reductions in BMI SDS (*F*(1,97) = 17.8, *p* < 0.001). Males achieved a reduction in BMI SDS *M* = 0.24 (*SD* = 0.08), a large effect size (*d* = 2.86, *95%CI*[2.10, 3.61]), and females achieved a reduction of *M* = 0.17 (*SD* = 0.08), also a large effect (*d* = 2.22, *95%CI*[1.76, 2.67]). There was no difference in overall BMI SDS or reduction over time between different school levels. There was no correlation between age and BMI SDS change.

#### Participants with baseline BMI SDS ≥ 3 vs participants with baseline BMI SDS < 3

Participants with baseline BMI SDS ≥ 3 achieved greater weight loss than those with BMI SDS < 3 (*F* (1, 97) = 18.4, *p* < 0.001). Participants with a baseline BMI SDS < 3 (*n* = 44) achieved a *M* = 3.80 kg (*SD* 1.38) reduction, a large effect (*d* = 2.75, *95%CI*[2.10, 3.40]). Those whose baseline BMI SDS ≥ 3 (*n* = 54) achieved a *M* = 5.87 kg (*SD* 2.67) reduction (*d* = 2.20, *95%CI*[1.70, 2.68]).

### Fatigue

With respect to PedsQL-MFS Total scores, for the full sample there was a significant reduction in fatigue over the 21 days (*t*(97) = 7.62, *p* < 0.001) of moderate effect size (*d* = 0.77, *95%CI*[0.54, 0.99]) (see Table 2). A mixed ANOVA of Time x Gender revealed a trend toward greater fatigue in females over both time points (*F*(1, 96) = 3.63, *p* = 0.143) but gender did not influence fatigue reduction over time (*F*(1, 96) = 2.29, *p* = 0.134). Similarly, there was no effect on fatigue from school grade level (*i.e.,* primary v secondary) (*F*(1, 96) = 0.68, *p* = 0.410) or effect of school level on changes in fatigue (*F*(1, 96) = 0.62, *p* = 0.434). Finally, BMI SDS level (≥ 3 vs < 3) did not influence fatigue (*F*(1, 95 = 1.67, *p* = 0.200) or its changes over time (*F* (1, 95) = 0.06, *p* = 0.80). There was no correlation between age and amount of change in any of the PedsQL-MFS total or subscales.

There were no significant differences between PedsQL-MFS subscale changes as indicated by confidence interval overlap: General Fatigue *d* = 0.73 [0.51, 0.95]; Sleep/Rest Fatigue *d* = 0.36 [0.16, 0.57]; Cognitive Fatigue *d* = 0.45 [0.24, 0.66]. Table 3 presents reliable change percentages for PedsQL Total and subscale scores. The majority did not experience a statistically significant change in fatigue symptoms, with 12–27% experiencing improvements in fatigue symptoms. A post-hoc exploration found that changes in BM were not correlated with changes in fatigue. Minor differences in rates of reliable improvement by gender or BMI SDS were not statistically significant.

**Table 3:** Reliable change analyses for pediatric quality of life multidimensional fatigue scale by gender and BMI SDS

	Group/subgroup	PedsQL-MFS total (%)	PedsQL-MFS sleep-rest (%)	PedsQL-MFS general (%)	PedsQL-MFS cognitive (%)
Reliable improvement	Male	13.9	16.7	13.9	8.3
	Female	34.4	23.4	28.1	14.1
	Total	27.0	21.0	23.0	12.0
	BMI SDS ≥ 3	25.5	23.6	23.6	9.1
	BMI SDS < 3	28.9	17.8	22.0	15.6
No reliable change	Male	83.3	80.6	83.3	88.9
	Female	62.5	68.8	68.8	84.4
	Total	70.0	73.0	74.0	86.0
	BMI SDS ≥ 3	70.9	70.9	70.9	87.3
	BMI SDS < 3	68.9	75.6	77.0	84.4
Reliable deterioration	Male	2.8	2.8	2.8	2.8
	Female	3.1	7.8	3.1	1.6
	Total	3.0	6.0	3.0	2.0
	BMI SDS ≥ 3	3.6	5.5	5.5	3.6
	BMI SDS < 3	2.2	6.7	0.0	0.0

BM = Body Mass; BMI SDS = Body Mass Index Standard Deviation Scores

### Youth self-report

Table 4 provides mean baseline and post-treatment scores on the YSR for the total sample. Only the attention problems subscale significantly improved, *t*(99) = 3.64, *p* < 0.001, *d* = 0.36 [0.16, 0.57] with 13% of the sample achieving reliable improvement. However, the change in YSR Attention Problems subscale did not significantly correlate with changes in Total PedsQL-MFS (*r* = 0.01, *p* = 0.905), Sleep/Rest Fatigue subscale (*r* = -0.01, *p* = 0.955), General Fatigue (*r* = 0.04, *p* = 0.702), Cognitive Fatigue (*r* = 0.00, *p* = 0.998), weight (*r* = -0.09, *p* = 0.374), SDS (*r* = -0.00, *p* = 0.966) or BMI SDS (*r* = -0.00, *p* = 0.966).

**Table 4:** Youth self-report scores at baseline and posttreatment

Subscale	Admission* M* (*SD*)	Discharge* M* (*SD*)	Paired-group t-test	% Reliably improved
Anxious/depressed	7.44 (5.34)	7.21 (5.27)	*t*(99) = 0.73, *p* = .469	3
Withdrawn/depressed	5.06 (3.68)	4.92 (3.63)	*t*(99) = 0.54, *p* = .589	5
Somatic complaints	4.76 (3.36)	4.61 (3.49)	*t*(98) = 0.75, *p* = .515	4
Social problems	5.25 (3.67)	4.98 (3.53)	*t*(99) = 1.03, *p* = .305	2
Thought problems	3.89 (3.83)	3.43 (3.36)	*t*(98) = 1.64, *p* = .104	4
Attention problems	7.08 (3.51)	6.19 (3.42)	*t*(99) = 3.64, *p* = < .001	13
Rule-breaking behaviour	4.49 (4.21)	4.85 (3.92)	*t*(99) = -1.11, *p* = .270	6
Aggressive behaviour	8.65 (5.62)	8.49 (4.85)	*t*(99) = 0.40, *p* = .688	9

BM = Body mass; BMI SDS = Body mass index standard deviation scores

## Discussion

When considering mean changes for the sample, significant reductions in BM, BMI SDS and fatigue were achieved during the 3-week inpatient multicomponent body weight reduction program. The magnitude of changes in BM were consistent with those achieved in previous evaluations of the 3-week BWRP [36, 37, 39, 51]. The combination of energy restriction to 75% resting energy expenditure combined with dietary education, daily personalised moderate physical exercise and counselling appears to be sufficient to achieve BM reductions of 3–7% in female and 4–9% in male children and adolescents with obesity, within 3 weeks. Previous studies demonstrated that reductions in BM occurred without reductions in Fat-Free Mass, and accompanied by increases in muscle strength [36, 39].

A previous study found the 3-week BWRP to reduce fatigue as measured by the Fatigue Severity Scale (FSS) [51], a brief self-report questionnaire. Encouragingly, FSS scores improved with no significant differences between male and female children and adolescents, or between those with and without metabolic syndrome. The present study is the first to demonstrate a similar level of improvement with a different measure (the PedsQL-MFS), increasing confidence in the consistency of the effect. In both studies, the mean reduction in fatigue was consistent across gender and ages, suggesting a robust effect. The present study builds on Rigamonti et al. [51] by exploring changes across multiple dimensions of fatigue. In the current sample, confidence intervals for the effect sizes of improvement in each fatigue dimension overlapped, suggesting the improvements in fatigue were consistent across dimensions. On the other hand, the effect size estimate was larger for general fatigue than sleep/rest or cognitive fatigue, suggesting that in a large sample with more precise estimates, different responses between dimensions might be expected. The potential to detect different degrees of improvement in different dimensions of fatigue is a primary advantage of a multidimensional measure like the PedsQL-MFS over shorter unidimensional measures. Recently, Boolani et al. [9] demonstrated that physical activity could increase physical energy and reduce physical fatigue after 8 h of sedentary behaviour, but did not increase mental energy or reduce mental fatigue. Different interventions may be required to alleviate different aspects of fatigue and multidimensional measures should better safeguard against missing important patient concerns. In the case of the 3-week BWRP, continued use might reveal that the program has stronger effects on physical fatigue but weaker effects on sleep/rest and cognitive fatigue. This could lead to focusing future program development on trialling new components targeting sleep/rest and cognitive fatigue symptoms, for example, like the addition of flaxseed to diets to reduce mental fatigue [23].

Because mean changes can mask the experience of individuals, this study – for the first time—examined the proportion of individuals making statistically reliable changes in fatigue. This revealed that a minority of the cohort was responsible for the majority of the cohort’s improvement in fatigue. Only a small proportion experienced a worsening of fatigue; the greatest extent was 6% who experienced a reliable worsening of sleep-related fatigue symptoms. Unfortunately, none of the participant characteristics measured in this study were clearly associated with reliable improvement in fatigue. Rates of improvement were similarly modest regardless of age, gender or baseline BMI SDS.

It is as yet unclear who is likely to experience an improvement in fatigue. [51] suggested a number of mechanisms by which fatigue might improve. One was due to increased agility through weight loss, although the present study does not support this as there was no relationship between changes in fatigue and BM changes. Other explanations were improvement in biochemical parameters and mood, but these were not measured in the current study. Future studies should seek to elucidate the mechanism by which BWRP leads to improvements in fatigue, given that it is a major obstacle to children and adolescents maintaining dietary and exercise regimes that continue and maintain weight loss.

The changes in weight and fatigue were generally not associated with changes in emotional and behavioural problems as detected by the Youth Self-Report, which showed only a significant improvement in attention problems for some individuals. Although only a minority showed reliable improvement, this is an intriguing and important effect as attention difficulties are associated with low academic achievement [35], social problems such as low acceptance, victimisation and rejection [31] and difficulties with self-managing medical conditions, such as type I diabetes management [60].

Although changes in attention were not correlated with changes in fatigue or body weight, they may be due to improvements in physical fitness after our multidisciplinary BWRP, as we have demonstrated to occur in a previous study performed on a comparable age- and BMI-matched obese population [36]. Alternatively, psychosocial aspects of the program such as its structure (multidisciplinary approach), close personal attention from empathic professionals (educators, psychologists, teachers at the Hospital school) and, last but not least, sense of relatedness with others with similar difficulties may have facilitated participants’ sense of attentional focus.

There were some limitations in the current study. Firstly, there was no control group so changes in fatigue cannot be conclusively attributed to the BWRP. Future studies should include a comparison group who did not receive the BWRP measured over the same time frame.

Secondly, post-intervention measures were only collected at discharge. Although it is useful to know the immediate effects of the intervention, the comparison is between a period preceding hospitalisation where patients are in their home environments and their regular routine with a period in the program with novel people and activities. It is difficult to predict how this may have affected responses. For example, participants may sleep better in their own homes than in a hospital and this may have attenuated the BWRP effect on sleep/rest fatigue. On the other hand, being in a supportive inpatient environment with children and adolescents with similar difficulties and away from the stigma of obesity may have improved participants’ “psychological” fatigue but in a way that is not sustainable in their everyday lives. Our previous experience has been that response rates to questionnaires administered post-discharge have been poor; however, future studies should explore incentives to improve response rates and follow-up participants during at least the following month post-discharge to provide a better indication of the effect of the BWRP on fatigue.

The present study included no measures of body composition which could have otherwise validated changes in BM and PedsQL-MFS. However, we have recently demonstrated in a large sample that the 3-week BWRP achieves a reduction in fat mass while preserving fat free mass and increasing lower limb power [36]. Together with Rigamonti et al.’s [51] findings that the reductions in fat mass while preserving fat free mass were associated with reductions in fatigue, it seems likely that similar body composition changes would have been achieved here.

A further limitation was that no other psychological measures, apart from the Youth Self-Report, were included. Depression [32], psychological distress [65] and recently, anxiety sensitivity [33] have been associated with greater fatigue severity in people with obesity. For this reason, to date we cannot rule out that psychological factors may influence the response in term of fatiguability of patients with obesity to our BWRP. Finally, the relatively small percentage of participants who made reliable changes in fatigue might reflect that the 3-week multidisciplinary BWRP (determining a 4.8% reduction of body weight) is too short for many individuals with obesity, who need prolonged periods of rehabilitation to achieve and sustain greater changes in BMI SDS.

The study also had several strengths. There was very high completion of the exercise and dietary components of the program, verified by professional staff. The sample was relatively large for a clinical population and included participants with a severe degree of obesity. It employed a measure of fatigue that has been validated in paediatric obesity populations.

Future studies should explore predictors of changes in fatigue associated with the 3-week BWRP and compare the 3-week program with longer programs to explore the strength of dose–effect relationships between the BWRP and fatigue changes. Future studies should also explore the efficacy of BWRP on fatigue in adults with obesity, investigating whether changes in weight loss and fatigue vary differentially with age.

## Conclusions

The 3-week BWRP achieved significant mean improvements in BM, BMI SDS and fatigue, however only a minority of individuals experienced reliable change in fatigue. Differences in the amount of improvement between dimensions of fatigue were not clearly distinct in this sample but are worth exploring in a larger sample. Future studies should also explore predictors of treatment responsiveness, including between dimensions of fatigue.

## Data Availability

The datasets used and/or analysed during the current study are available from the corresponding author on reasonable request.
